# Complete Genome Sequence of an Influenza C Virus Strain Identified from a Sick Calf in the United States

**DOI:** 10.1128/MRA.00828-18

**Published:** 2018-07-12

**Authors:** Hewei Zhang, Elizabeth Poulsen Porter, Molly Lohman, Nanyan Lu, Lalitha Peddireddi, Gregg Hanzlicek, Douglas Marthaler, Xuming Liu, Jianfa Bai

**Affiliations:** a Kansas State Veterinary Diagnostic Laboratory, College of Veterinary Medicine, Kansas State University, Manhattan, Kansas, USA; b Department of Diagnostic Medicine and Pathobiology, College of Veterinary Medicine, Kansas State University, Manhattan, Kansas, USA; c State Key Laboratory for Molecular Biology of Special Economic Animals, Institute of Special Economic Animal and Plant Sciences, Chinese Academy of Agricultural Sciences, Changchun, China; Queens College

## Abstract

Influenza C virus (ICV) has been identified for the first time from bovine respiratory disease complex (BRDC) samples in the United States. Here, we report the complete genome sequence of the strain C/bovine/Montana/12/2016, identified from a nasal swab sample collected from a sick calf with clinical signs of respiratory disease in Montana.

## ANNOUNCEMENT

Influenza viruses, including influenza A, B, C, and D viruses, are contagious zoonotic pathogens that can cause influenza and may be transmitted among animals and humans (1–3). Influenza C virus (ICV) was first identified in humans in 1947, and it was originally thought to be exclusively a human pathogen until it was also identified in pigs in China (4, 5). Recently, we identified ICV from bovine respiratory disease complex (BRDC) samples in the United States. A total of 1,525 BRDC diagnostic samples were collected from 2016 to 2018 and screened with an ICV reverse transcription-quantitative PCR (RT-qPCR). Sixty-four ICV-positive samples mainly from the midwestern United States were identified, among which 12 were confirmed by sequencing a 590-bp fragment of the ICV matrix gene using primers ICV-cF (AAAGCCAGCACAGCAATGAA) and ICV-cR (TCAAAAATACCATCATTGGAAAAAGG).

A complete genome sequence has been generated from C/bovine/Montana/12/2016 virus, which was identified from a nasal swab sample collected in Montana in November 2016 from a sick calf with clinical signs of respiratory disease. Viral RNA was extracted from the bovine clinical sample using a QIAamp viral RNA minikit (Qiagen, Valencia, CA). Single-reaction genomic amplification of ICV segments from viral RNA was performed using a SuperScript IV one-step RT-PCR system (Invitrogen/ThermoFisher, Carlsbad, CA) as previously described (6, 7). The primers contain the conserved ICV RNA termini (underlined) and a 5′ tail for efficient amplification of all ICV RNA segments, ICV-3uniPlusF (5ʹ-ACGCGTGATCAGCAGAAGCAGG-3ʹ) and ICV-5uniPlusR (5ʹ-ACGCGTGATCAGCAGTAGCAAG-3ʹ). ICV genes were further amplified with a TaKaRa LA *Taq* PCR kit (TaKaRa, Mountain View, CA) using ICV gene-specific primers (8, 9). The amplified gene fragments were sequenced at Genewiz (South Plainfield, NJ). The sequences were assembled using CLC Genomic Workbench 9.0.1 (CLC Bio/Qiagen, Cambridge, MA).

The complete coding sequence (CDS) lengths of the seven segments, including polymerase basic 2 (PB2), PB1, polymerase 3 (P3), hemagglutinin-esterase (HE), nucleoprotein (NP), matrix (M), and nonstructural (NS) genes, are 2,325, 2,265, 2,130, 1,944, 1,698, 1,125, and 862 nucleotides, respectively. The 7 genes code for 9 viral proteins, namely PB2, PB1, P3, HE, NP, M1, CM2, NS1, and NS2, with lengths of 774, 754, 709, 647, 565, 242, 139, 246, and 182 amino acids, respectively. Phylogenetic analysis revealed that the C/bovine/Montana/12/2016 virus was most closely related to the human ICV strain C/Mississippi/80, with overall genome sequence identity of 97.1% and specifically 97.0% for the PB2 gene, 97.7% for the PB1 gene, 97.5% for the P3 gene, 96.2% for the HE gene, 96.8% for the NP gene, 96.8% for the M gene, and 97.6% for the NS gene.

This is the first report of full-genome information of an ICV strain identified from bovines. Although interspecies transmission of influenza viruses occurs among animals and humans and a high concentration of ICV was identified in a sick calf, more detailed investigations are needed to confirm if ICV is involved in bovine respiratory disease and to illustrate the zoonotic potential of bovine ICV strains to cause human disease.

### Data availability.

The complete genome sequence of the C/bovine/Montana/12/2016 virus was deposited in GenBank as 7 individual segments under consecutive accession numbers from MH348113 to MH348119.
